# Positive Preemptive Analgesia Effectiveness of Pregabalin Combined with Celecoxib in Total Knee Arthroplasty: A Prospective Controlled Randomized Study

**DOI:** 10.1155/2023/7088004

**Published:** 2023-01-12

**Authors:** Yi Zhou, Xiaoyan Liu, Chuan Ding, Bingyan Xiang, Ling Yan

**Affiliations:** ^1^The Department of Orthopaedics, The Jian Yang Hospital of Traditional Chinese Medicine, Jianyang 641400, China; ^2^The Department of Orthopaedics, The Third Affiliated Hospital of Zunyi Medical University (The First People's Hospital of Zunyi City), Zunyi 563000, China

## Abstract

**Objective:**

The purpose of the present study (a randomized clinical trial) was to evaluate the preemptive analgesic effects of pregabalin combined with celecoxib in total knee arthroplasty (TKA).

**Methods:**

From January 2019 to June 2021, we enrolled 149 patients who underwent TKA and divided them into four groups: the placebo group (*n* = 36), celecoxib group (*n* = 38), pregabalin group (*n* = 38), and combination group (*n* = 37). Each group was given the corresponding preemptive analgesia regimen at 12 and 2 hours before surgery. The pain score at rest and upon movement, cumulative dosage of sufentanil, knee range of motion (ROM), high-sensitivityC-reactive protein (hs-CRP) level, and adverse effects were evaluated after TKA to compare the effects of the preemptive analgesia regimens among the four groups.

**Results:**

The pain scores upon movement were significantly lower in the combination group than in the other three groups at 6, 12, 24, and 48 hours after surgery (*P* < 0.05). The cumulative dose of sufentanil within 48 hours after surgery was lowest in the combined group among the four groups (*P* < 0.05). Hs-CRP, ROM, and postoperative nausea and vomiting (PONV) were within 72 hours after surgery significantly improved in the combination group compared with those of the three other groups (*P* < 0.05).

**Conclusion:**

The preemptive analgesia regimen of pregabalin combined with celecoxib had positive effects on improving acute pain and reducing the cumulative dose of opioids after TKA. This trial is registered with ChiCTR2100041595.

## 1. Introduction

Total knee arthroplasty (TKA), a surgical method for the treatment of terminal stage knee osteoarthritis, can effectively relieve pain and restore knee function. However, patients experience moderate to severe pain after TKA. Acute pain after TKA can have a negative effect on enhanced recovery after surgery among patients [1]. If acute postoperative pain is not well managed, it may seriously affect the patient's knee functional recovery, sleep, and cardiopulmonary function and prolong the hospital stay, and it may even develop into uncontrollable chronic pain, which can seriously affect the patient's quality of life [2].

The mechanism of postoperative pain is complicated. In the past, it was believed that postoperative pain after TKA was mainly nociceptive pain caused by surgical trauma and tissue inflammation [3]. However, recent studies have shown that tissue inflammatory mediators can repeatedly stimulate pain sensory nerves, leading to a large sensitization of pain sensory nerves and resulting in neuropathic pain [4, 5]. Therefore, postoperative pain after TKA is a mixed pain involving multiple mechanisms, and the analgesic effect of a single drug is often not sufficient. At present, multimodal analgesia is considered to be an effective treatment for postoperative analgesia after TKA [6]. It combines analgesic drugs of different mechanisms to exert a synergistic or additive effect on analgesia while reducing the single drug dose and adverse effects. Preemptive analgesia is an important part of multimodal analgesia, which means that various analgesic measures are adopted in advance to inhibit the sensitization of peripheral and central pain nerves. It can improve the pain threshold of patients and reduce the degree of pain experienced [7]. Many studies have demonstrated the positive effectiveness of preemptive analgesia in TKA [8–10].

The use of oral analgesics in advance is a convenient and effective method for preemptive analgesia after TKA. Celecoxib or other cyclooxygenase-2 (COX-2) inhibitors selectively inhibit the activity of COX-2, reduce prostaglandin production and inflammatory reactions, and achieve effective control of pain and anti-peripheral pain sensitization effects. In particular, it has a positive effect on reducing nociceptive pain mediated by inflammatory mechanisms after TKA [11, 12]. Many previous studies reported that celecoxib was effective in preemptive analgesia during TKA [13–15]. Pregabalin is a latest-generation analog of the inhibitory neurotransmitter *γ*-aminobutyric acid (GABA). Different from the mechanism of traditional analgesics, it has obvious anti-central and peripheral pain sensitization effects through inhibiting the voltage-dependent calcium channels of the peripheral and central nervous systems [16]. Therefore, it can improve the pain threshold and be used to control neuropathic pain. Because of the stable pharmacokinetics of pregabalin and few adverse effects, it has attracted more attention as a new type of analgesic drug in TKA. Recent studies have reported preemptive analgesic effects of pregabalin in TKA [17, 18]. Theoretically, the combination of two different analgesic mechanisms can improve the analgesic effect. However, whether the combination of celecoxib and pregabalin can further improve analgesia after TKA remains unknown. To our knowledge, there are currently few clinical studies combining these two drugs for the preemptive analgesia of TKA.

The purpose of our study was to evaluate the effectiveness of pregabalin combined with celecoxib as TKA preemptive analgesia by compared the pain score, cumulative dosage of opioids, high-sensitivity C-reactive protein (hs-CRP) level, and adverse effects after TKA. We hypothesised that the effectiveness of pregabalin combined with celecoxib as TKA preemptive analgesia would lead to satisfactory clinical outcomes.

## 2. Materials and Methods

### 2.1. Ethical Approval

The present study was a double-blind randomized clinical trial and was conducted in the affiliated hospital of our university. The study was approved by the ethics committee of the affiliated hospital of our university and conducted in accordance with the Declaration of Helsinki. In addition, the study was retrospectively registered at http://www.chictr.org.cn (ChiCTR2100041595, January 1, 2021). All patients signed written informed consent forms.

### 2.2. Inclusion and Exclusion Criteria

From January 2019 to June 2021, 201 patients who underwent TKA were subjected to a strict eligibility review. The inclusion criteria were as follows: patients who received elective, initial, and single TKA. The exclusion criteria were as follows: (1) patients with an American Society of Anesthesiologists (ASA) classification of grade 4 or higher, (2) patients with a history of liver and kidney insufficiency, severe cardiopulmonary disease, severe digestive tract disease, and mental illness, (3) patients with an allergy to celecoxib or pregabalin or to anesthetic drugs, (4) patients who had taken celecoxib or pregabalin within 2 weeks before surgery, and (5) patients with cognitive impairment. A total of 160 patients were finally included in the study.

### 2.3. Design and Conduct of the Study

Pregabalin and celecoxib are produced by Pfizer in the United States under the trade names Lyrica and Celebrex, respectively. Pregabalin, celecoxib, and the placebo were packaged in the same opaque packaging by the University Hospital Health Research Center pharmacy. The placebo capsule contained a mixture of 50% cellulose and 50% starch. An assistant from the Clinical Research Center used a random number table to divide the patients into four groups: the control group, celecoxib group, pregabalin group, and combination group. The patient group assignments were sealed in envelopes. Anesthesiologists who did not participate in the study opened the envelope 12 hours before the surgery, distributed drugs according to the patient group assignment, and distributed the same drugs again 2 hours before the surgery. The researchers involved did not know the patient group assignment during the surgery or data analysis. The medication program was as follows: placebo group: 200 mg + 150 mg placebo; pregabalin group: 150 mg pregabalin + 200 mg placebo; celecoxib group: 200 mg celecoxib + 150 mg placebo; and pregabalin combined with celecoxib group: 150 mg pregabalin + 200 mg celecoxib. Each group was given the same medication program again 2 hours before surgery. The patients were closely monitored for adverse effects after each administration of the medicine. All patients were given general anesthesia by senior anesthesiologists in our hospital. All operations were performed by two senior doctors with more than ten years of experience in TKA. Periarticular infiltration of the cocktail ingredient (0.5% ropivacaine + 10 mg/ml triamcinolone acetonide acetate + 0.1% epinephrine hydrochloride) was performed before closed the incision. Afterwards, the four groups of patients received patient-controlled intravenous analgesia (PCIA) immediately after the laryngeal mask was removed. The analgesic formula was sufentanil 2 *μ*g/kg + ondansetron 0.3 mg/mL + 0.9% sodium chloride injection, which were diluted to 100 mL. The first loading dose was 3 mL; there was no background dose. The additional dose of self-controlled analgesia was 1 mL each time, the lock-in time was 15 min, and the use of PCIA lasted for 48 hours. After the surgery, if the patients had a visual analog scale (VAS) score >4, 10 mg morphine was given as remedial analgesia, and none of the patients underwent a nerve block. All patients were managed with a standardized TKA accelerated rehabilitation program, and immediately after the operation, they were instructed to perform lower limb quadriceps muscle contraction exercises, lower limb ankle pump exercises, and active and passive knee flexion activities.

The main observation indicators included the following:

(1) VAS pain score at rest and upon movement at 6, 12, 24, and 48 hours after the surgery (from 0: no pain to 10: worst possible pain; pain upon movement was pain upon maximum flexion of the knee); (2) cumulative dose of opioids within 48 hours (including total sufentanil and morphine remedial analgesic dosages, all converted to sufentanil equivalent); (3) the hs-CRP level before and after the surgery; (4) time of first successful straight leg elevation: the standard is that the patient can maintain a flexed ankle joint and straight leg raise at least 40 cm above the bed for more than 4 s; (5) maximum knee flexion range of motion (ROM) at 24, 48, and 72 hours after surgery; (6) time to first remedial analgesia after surgery; and (7) complications including postoperative nausea and vomiting (PONV), dizziness, gastrointestinal hemorrhage, urinary retention, deep vein thrombosis (DVT), and severity of sedation.

### 2.4. Statistical Analysis

Power analysis was performed based on the data from a previous study [18] to calculate the sample size (PASS software, version 16.0; USA). To show a 30% reduction in sufentanil consumption, with a power of 80% and an alpha of 5%, each group requires 25 patients, and the 4 groups require 100 patients. Considering a 20% loss to follow-up rate, we recruited 160 patients, and 149 were included in the final data analysis. The data were analyzed using SPSS 26.0 statistical software (version 26.0; IBM, USA). The normality assessment of continuous numerical variables was analyzed with the Kolmogorov–Smirnov test. Continuous measurement data with normal distributions are represented by the mean ± standard deviation; otherwise, the median plus the interquartile range is used. Categorical variables are represented by the quantity and percentage. Repeated measures ANOVA was used for the repeated measures indicators (opioid cumulative dosages, postoperative pain scores, hs-CRP level, and ROM), and a simple effects model was used to explore the interaction between time and group. The Bonferroni method was used to adjust the test level. Linear regression was used to explore the relationship between postoperative pain scores and the cumulative dosage of sufentanil. Chi-square, one-way ANOVA, Kruskal–Wallis, and Fisher exact probability tests were used as appropriate. *P* < 0.05 indicated that the difference was statistically significant.

## 3. Results

**Figure 1:**
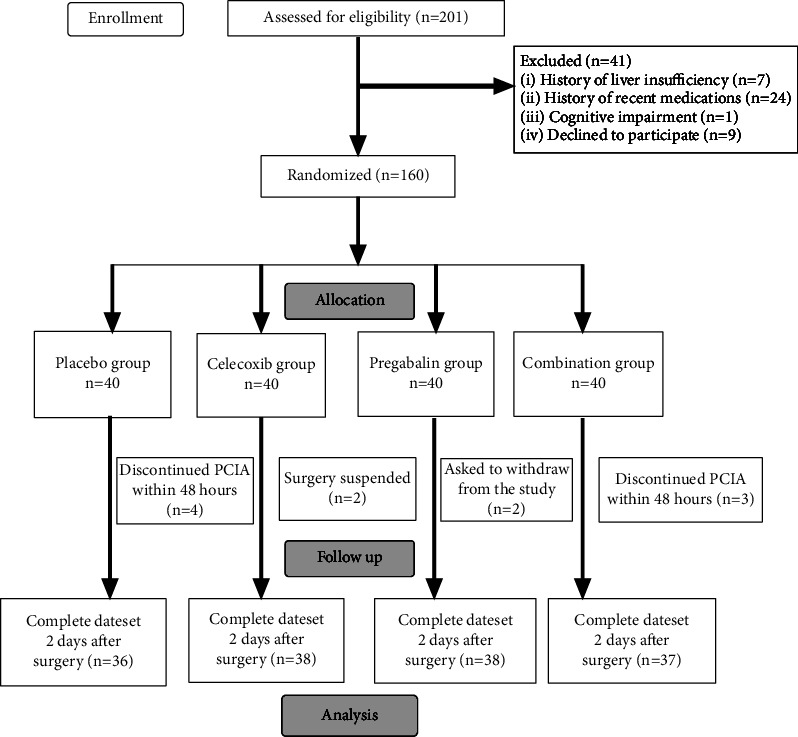
The CONSORT study participant flow diagram.

A total of 160 patients were enrolled in the study, 7 patients discontinued PCIA because of severe vomiting reaction to sufentanil. 2 patients asked to withdraw from the study and 2 patients were suspended surgery. Consequently, 149 patients were included in the final data analysis. The CONSORT study participant flow diagram is shown in Figure 1.

### 3.1. Patient and Surgical Characteristics

There were no significant differences in the patient and surgical characteristics among the four groups (*P* > 0.05) (Table 1).

### 3.2. VAS Pain Scores and the Cumulative Dose of Sufentanil

**Figure 2:**
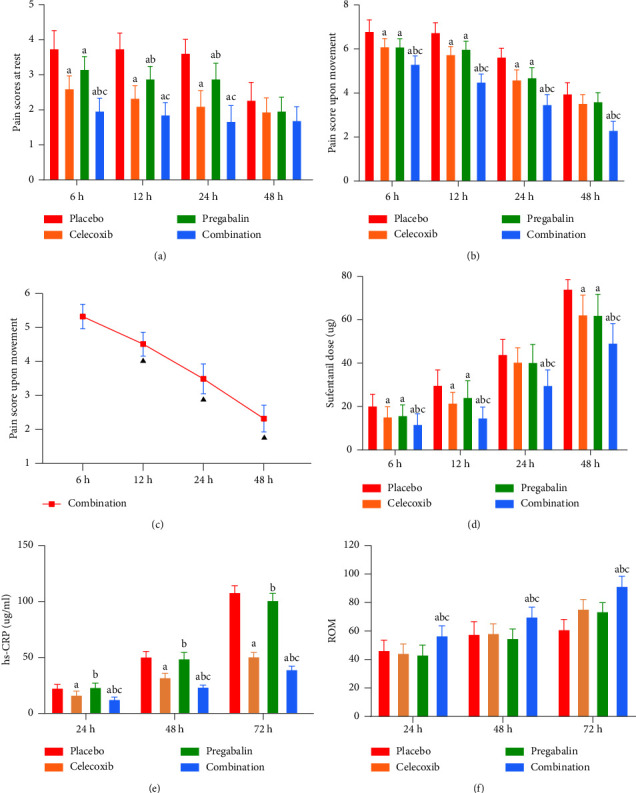
(a) Pain score at rest. (b) Pain score upon movement. (c) Pain score upon movement in the combination group. (d) Cumulative dose of sufentanil. (e) High-sensitivity C-reactive protein (hs-CRP). (f) Knee range of motion (ROM). ▲ indicates a significant difference compared with a previous time point in the combination group, which suggests that the pain scores upon movement in the combined group significantly decreased continuously over time. a indicates a significant difference compared with the placebo group (*P* < 0.05); b indicates a significant difference compared with the celecoxib group (*P* < 0.05); c indicates a significant difference compared with the pregabalin group (*P* < 0.05). The difference was significant at the (*P* < 0.05) level.

As shown in Table 2 and Figure 2, the pain scores at rest in the combination group were significantly lower than those in the pregabalin group and the placebo group at 6, 12, and 24 hours after surgery (*P* < 0.05). The pain scores at rest in the combination group were significantly lower than those in the celecoxib group at 6 hours after surgery (*P*=0.018 < 0.05) (Figure 2(a)). The pain scores upon movement in the combination group were significantly lower than those of the other three groups at 6, 12, 24, and 48 hours after surgery (*P* < 0.05) (Figure 2(b)). There was an interaction between time and group in the combined group (*F*_(3,145)_ = 37.88, *P* < 0.001). Then, a simple effects model was used for further analysis. In terms of a time effect, we observed that the pain score on movement in the combined group showed a significantly continuous downward trend over time within 48 hours after surgery (*P* < 0.05) (Figure 2(c)). The cumulative dose of sufentanil in the combination group (49.30 ± 10.91 *µ*g) was significantly lower than those of the other three groups within 48 hours after surgery (*P* < 0.05) (Figure 2(d)). The first rescue analgesic time of the combined group (203.89 ± 10.87 min) was significantly longer than those of the other three groups (*P* < 0.05).

### 3.3. hs-CRP Level, Knee Function Indicator, and Incidence of Postoperative Complications

As shown in Table 3 and Figure 2, there was no significant difference in hs-CRP levels among the four groups before surgery (*P*=0.386). The hs-CRP level was significantly lower in the combination group than in the other three groups at 24, 48, and 72 hours after surgery (*P* < 0.05). The hs-CRP level was significantly lower in the celecoxib group than that in the pregabalin and placebo groups at 24, 48, and 72 hours after surgery (*P* < 0.05) (Figure 2(e)). ROM increased significantly in the combination group compared with the three other groups at 24, 48, and 72 hours after surgery (*P* < 0.05) (Figure 2(f)). The time of the first straight leg elevation test was significantly shorter in the combined group (17.98 ± 2.09 h) than in the other three groups (*P* < 0.05). As shown in Table 4, the incidence of postoperative nausea and vomiting (PONV) was significantly lower in the combination group than in the other three groups (*P* < 0.05).

### 3.4. Linear Regression Analysis

**Figure 3:**
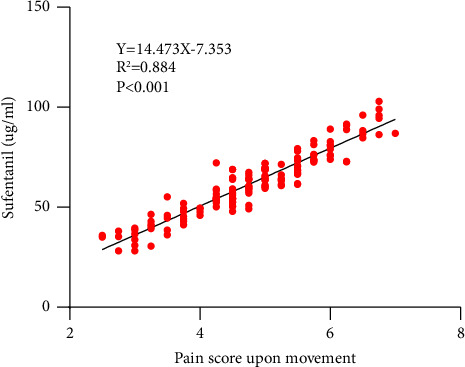
Linear regression analysis. The mean pain score upon movement within 48 hours after surgery was the independent variable (*X*). The cumulative dose of sufentanil within 48 hours after surgery was the dependent variable (*Y*). The difference was significant at the *P* < 0.05 level.

In the linear regression analysis, the mean pain score upon movement within 48 hours after surgery (4.78 ± 1.02) was the independent variable (*X*), and the cumulative dose of sufentanil within 48 hours was the dependent variable (*Y*). The regression equation (*Y* = 14.743*X* − 7.353, *R*^2^ = 0.884, *F* = 51.661, *P* < 0.001) (Figure 3) suggested that for every 1-point decrease in the mean pain score upon movement within 48 hours after surgery, the cumulative dose of sufentanil will decrease by 14.743 ug.

## 4. Discussion

Perioperative pain management is key to the smooth implementation of enhanced recovery after surgery (ERAS), which has rapidly become a popular concept in the field of orthopedics. Although the combined application of various postoperative analgesia methods can improve the postoperative acute pain of TKA patients, many still suffer from moderate or even severe pain [19, 20].

Preemptive analgesia is an important part of multimodal analgesia, and its value is increasingly recognized by clinicians and researchers. Some studies have found that preemptive analgesia can further improve postoperative pain in TKA patients [8–10]. However, the optimal preemptive analgesia regimen for TKA is controversial.

In the present study, we observed that the combination group had better improvement in pain scores at rest and upon movement than the other three groups. The pain scores at rest in the combined group were always low level within 48 hours, and unlike the placebo group, there were no large fluctuations in pain scores, which indicated that the analgesic effect of the combination regimen was stable and relatively durable. However, the pain upon movement was more significant than that at rest because all of the patients need to exercise as soon as possible after TKA to enhance recovery. In the study of Lee et al. [21], patients used 150 mg pregabalin and 200 mg celecoxib 2 hours before TKA, and the pain scores upon movement at 6, 12, 24, and 48 hours after the surgery were significantly lower than those of the control group. Kien et al. [22] used 150 mg pregabalin and 200 mg celecoxib preoperatively for lumbar spine surgery patients and observed that the pain score upon movement was significantly lower than that of the control group within 48 hours after surgery; this result is consistent with our study. The pain scores upon movement at 6, 12, 24, and 48 hours after surgery were significantly lower in the combination group than in the other three groups. Furthermore, we demonstrated that the pain score upon movement decreased continuously over the first 48 hours in the combination group, while such a trend was not observed in the other groups. This positive trend may have a positive impact on early functional exercise in patients after TKA. Postoperative functional exercise involves greater pain sensitization compared with rest, and a large number of inflammatory mediators are released during functional exercise. The combined application of celecoxib and pregabalin had positive effects on the control of peripheral hyperalgesia and central hyperalgesia and cascading effects on inflammatory mediator release. Therefore, we believe that the analgesic regimen of pregabalin combined with celecoxib may have more practical application value in TKA preemptive analgesia than a single drug.

Currently, analgesia after TKA mainly involves a variety of opioids, such as sufentanil, tramadol, and morphine. Opioids mainly act on opioid receptors in the pain center of the brain to inhibit the generation and amplification of pain signals. However, opioids have significant side effects, such as nausea and vomiting, drug dependence, urinary retention, excessive sedation, and respiratory depression. The majority of TKA patients are elderly patients, and the incidence of opioid side effects is significantly higher in elderly patients than in young and middle-aged patients [23]. One of the goals of multimodal analgesia is to reduce the amount of opioids to improve postoperative complications and drug addiction [24]. The cumulative dose of sufentanil and the incidence of PONV within 48 hours after surgery were significantly lower in the combination group than in the other three groups. This may suggest that the opioid-sparing effects of the preemptive analgesic regimen of pregabalin combined with celecoxib can reduce the incidence of postoperative complications. Furthermore, the linear regression analysis results showed that for every 1-point decrease in the mean pain score upon movement within 48 hours after surgery, the cumulative dose of sufentanil will decrease by 14.743 ug. This result suggests that if an effective preemptive analgesia regimen is used before TKA, the pain scores upon movement will be controlled at a low level, which would reduce the cumulative dose of opioids after TKA and consequently opioid side effects.

The key core of preemptive analgesia is to reduce the degree of peripheral and central pain sensitization and to improve the pain threshold of patients in advance [25]. Lower levels of inflammatory factors are strongly correlated with reduced peripheral and central pain sensitization [26]. hs-CRP levels at 24, 48, and 72 hours after surgery were significantly lower in the combination group than in the other three groups. An animal experiment performed by Bannister et al. [26] demonstrated that pregabalin reduces central pain sensitization by reducing the release of spinal pain neurotransmitters and inhibiting the excitability of the pain nerve center. On the other hand, several studies have reported that celecoxib improves the levels of hs-CRP by inhibiting inflammatory reactions [11, 13, 27]. In a clinical study by Jianda et al. [28], 400 mg celecoxib was given 2 hours before surgery to patients undergoing TKA. They found that the hs-CRP level in the preemptive analgesia group was significantly lower than that in the control group within one week after surgery. Therefore, we suggest that celecoxib and pregabalin may have a synergistic effect on the control of body inflammation levels. However, because inflammatory and pain sensitization mechanisms involve multiple links and multiple factors, further research is needed.

Our study has some limitations. First, our research was limited to within 72 hours after surgery, so we cannot be sure whether control of acute postoperative pain can promote long-term knee function recovery and reduce the possibility of long-term chronic pain. Second, we did not accurately estimate sample size, which may affect the efficacy of the present study. Third, according to the relevant study [29], the minimal clinically important differences (MCIDs) of pain was defined as 1.3 score at rest and 1.5 score during motion on a 0–10 VAS score. Therefore, only the pain upon movement at 12 hours of combination group reached the MCIDs compared the other three groups.

Last, in terms of postoperative complications, our sample size was too small to comprehensively and objectively evaluate the safety of analgesia programs.

## 5. Conclusion

The preemptive analgesia regimen of pregabalin combined with celecoxib had positive effects on improving acute pain and reducing the cumulative dose of opioids after TKA.

## Figures and Tables

**Table 1:** General characteristics of the patients.

	Placebo (*n* = 36)	Celecoxib alone (*n* = 38)	Pregabalin alone (*n* = 38)	Combination (*n* = 37)	Statistics	*P* value
Age, years	62.6 ± 7.9	61.5 ± 7.1	64.9 ± 7.7	63.0 ± 6.5	*F* = 1.385	0.251
Sex, male/female	9/27	10/28	8/30	8/29	*χ * ^2^ = 0.412	0.938
BMI	24.7 ± 3.8	24.9 ± 4.1	25.3 ± 4.3	25.4 ± 4.1	*F* = 0.245	0.865
Duration of surgery (min)	883 ± 22.1	84.6 ± 23.2	84.6 ± 27.2	89.9 ± 22.6	*F* = 0.326	0.806
ASA (I/II/III)	2/24/10	1/25/12	3/27/8	1/23/13	*χ * ^2^ = 3.188	0.801
Preoperative pain upon movement (VAS)	6.15 ± 1.14	6.51 ± 1.03	6.69 ± 1.23	6.36 ± 1.17	*F* = 1.501	0.217
Preoperative pain at rest (VAS)	3.03 ± 1.26	3.30 ± 1.17	3.39 ± 1.42	3.28 ± 1.18	*F* = 0.564	0.640
HSS score	41.5 ± 4.5	40.3 ± 3.8	39.5 ± 4.5	39.2 ± 4.3	*F* = 2.092	0.104

ASA, American Society of Anesthesiologists. HSS score, hospital for special surgery rating scale. VAS, visual analog scale (0: no pain to 10: worst possible pain); Pain upon movement: pain upon maximum flexion. Difference is significant at the *P* < 0.05 level.

**Table 2:** VAS pain scores and the cumulative dose of sufentanil.

	Placebo (*n* = 36)	Celecoxib alone (*n* = 38)	Pregabalin alone (*n* = 38)	Combination (*n* = 37)	*F*	*P* value
*Pain at rest (VAS)*						
Pain at rest at 6 hours	3.75 ± 0.97	2.61 ± 0.76^a^	3.16 ± 1.01^a^	1.97 ± 0.90^abc^	25.592	0.0001
Pain at rest at 12 hours	3.75 ± 0.84	2.34 ± 0.75^a^	2.89 ± 0.89^ab^	1.86 ± 0.67^ac^	38.050	0.0001
Pain at rest at 24 hours	3.62 ± 0.89	2.11 ± 0.95^a^	2.89 ± 1.01^ab^	1.68 ± 0.78^ac^	32.604	0.0003
Pain at rest at 48 hours	2.28 ± 1.00	1.95 ± 1.06	1.97 ± 0.97	1.70 ± 0.78	2.196	0.091

*Pain upon movement (VAS)*						
Pain upon movement at 6 hours	6.81 ± 1.11	6.11 ± 1.11^a^	6.10 ± 1.25^a^	5.32 ± 1.08^abc^	10.414	0.0001
Pain upon movement at 12 hours	6.75 ± 1.16	5.76 ± 1.08^a^	6.00 ± 1.27^a^	4.51 ± 1.15^abc▲^	23.312	0.0004
Pain upon movement at 24 hours	5.64 ± 1.42	4.61 ± 1.18^a^	4.71 ± 1.21^a^	3.49 ± 0.96^abc▲^	19.755	0.0003
Pain upon movement at 48 hours	3.97 ± 0.91	3.54 ± 0.76	3.62 ± 0.96	2.32 ± 0.82^abc▲^	3.336	0.021

*Sufentanil cumulative dose (ug)*						
Postoperative 6 hours	20.38 ± 5.24	15.29 ± 4.63^a^	15.90 ± 4.80^a^	11.83 ± 4.81^abc^	18.945	0.0002
Postoperative 12 hours	29.93 ± 8.89	21.70 ± 6.90^a^	24.30 ± 7.57^a^	14.90 ± 4.87^abc^	27.500	0.0002
Postoperative 24 hours	44.08 ± 11.83	40.53 ± 9.51	40.42 ± 11.17	29.84 ± 7.02^abc^	13.832	0.0001
Postoperative 48 hours	74.18 ± 15.29	62.38 ± 12.97^a^	62.17 ± 13.51^a^	49.30 ± 10.91^abc^	21.466	0.0003
Time for requirement of first dose of rescue analgesic (min)	89.41 ± 9.19	109.56 ± 11.90^a^	107.10 ± 12.13^a^	203.89 ± 10.87^abc^	79.763	0.0002

VAS, visual analog scale (0: no pain to 10: worst possible pain); pain upon movement: pain upon maximum flexion; difference was significant at the *P* < 0.05 level. a means there was a significant difference compared with the placebo group (*P* < 0.05). b means there was a significant difference compared with the celecoxib group (*P* < 0.05). c means there was a significant difference compared with the pregabalin group (*P* < 0.05). ▲ means there was a significant difference compared with a previous time point in the combination group, suggesting that the pain scores upon movement in the combined group decreased continuously over time.

**Table 3:** hs-CRP level and knee functional indicator.

	Placebo (*n* = 36)	Celecoxib alone (*n* = 38)	Pregabalin alone (*n* = 38)	Combination (*n* = 37)	*F*	*P* value
*hs-CRP (ug/mL)*						
Preoperative	1.96 ± 0.47	2.08 ± 0.55	2.06 ± 0.45	2.17 ± 0.50	1.019	0.386
Postoperative 3 hours	23.05 ± 2.92	16.82 ± 3.16^a^	24.62 ± 4.60^b^	12.03 ± 1.73^abc^	26.791	0.0002
Postoperative 24 hours	50.85 ± 4.60	32.46 ± 3.53^a^	49.20 ± 5.37^b^	23.93 ± 2.19^abc^	33.442	0.0002
Postoperative 48 hours	108.43 ± 5.84	51.10 ± 3.52^a^	101.30 ± 6.08^b^	39.55 ± 2.89^abc^	56.184	0.0001

*ROM (degrees)*						
Postoperative 24 hours	46.65 ± 6.91	44.71 ± 7.63	43.49 ± 9.41	56.91 ± 5.21^abc^	25.059	0.0002
Postoperative 48 hours	57.94 ± 8.43	58.58 ± 7.78	55.04 ± 8.89	70.12 ± 6.82^abc^	25.896	0.0001
Postoperative 72 hours	61.27 ± 6.37	75.58 ± 7.77	73.90 ± 9.55	91.73 ± 7.77^abc^	44.677	0.003
Time of first straight leg raise (hours)	21.83 ± 3.47	20.40 ± 2.04	20.14 ± 3.17	17.98 ± 2.09^abc^	12.079	0.0003

hs-CRP, high-sensitivity C-reactive protein; ROM, knee range of motion. a means there was a significant difference compared with the placebo group (*P* < 0.05). b means there was a significant difference compared with the celecoxib group (*P* < 0.05). c means there was a significant difference compared with the pregabalin group (*P* < 0.05).

**Table 4:** Incidence of postoperative complications.

	Placebo (*n* = 36)	Celecoxib alone (*n* = 38)	Pregabalin alone (*n* = 38)	Combination (*n* = 37)	*χ* ^2^	*P* value
PONV	8 (22.2%)	6 (15.8%)	5 (13.1%)	0 (0.00%)	8.483	0.034
Sedation score > 2	0 (0.00%)	0 (0.00%)	2 (5.26%)	2 (5.41%)	3.328	0.332
Dizziness	1 (2.78%)	1 (2.63%)	1 (2.63%)	2 (5.41%)	5.745	0.654
Urinary retention	1 (2.78%)	1 (5.26%)	4 (10.53%)	3 (8.11%)	1.216	0.876
Deep vein thrombosis	1 (2.78%)	1 (2.63%)	2 (5.26%)	0 (0.00%)	2.050	0.752
Gastrointestinal hemorrhage	0 (0.00%)	0 (0.00%)	0 (0.00%)	0 (0.00%)	—	—

Sedation was scored as 0 = no sedation; 1 = intermittent drowsiness; 2 = patient drowsy but arousable to verbal stimulus; and 3 = impossible to arouse verbally. PONV: postoperative nausea and vomiting; the difference was significant at the *P* < 0.05 level.

## Data Availability

The data used to support the findings of this study are available from the corresponding author upon request.

## Supplementary Materials

Supplementary Materials Supplementary material: the supplementary material was the raw data of this study.
