# Supplementary material for: Positive Preemptive Analgesia Effectiveness of Pregabalin Combined with Celecoxib in Total Knee Arthroplasty: A Prospective Controlled Randomized Study
Source: Pain Res Manag. 2023 Jan 12;2023:7088004. doi: 10.1155/2023/7088004 (PMC9851777; doi:10.1155/2023/7088004)
Supplement: Supplementary Materials — Supplementary material: the supplementary material was the raw data of this study. [file 7088004.f1.pdf]

病人编号	住院号	组别	年龄	性别	体重指数	手术时间	热痛阈值差值
1	1902110169	1	75	女	30.21	120	1.51
2	1901090306	1	59	女	23.15	79	1.12
3	1811240081	1	65	女	25.97	96	1.27
4	1811060172	1	53	女	20.32	63	0.96
5	1810090079	1	72	女	28.92	113	1.44
6	1810310207	1	65	女	25.91	95	1.27
7	1810120175	1	54	女	20.92	66	0.99
8	1907150079	1	52	女	18.44	52	0.86
9	1907150045	1	77	女	31.94	130	1.60
10	1907100323	1	61	女	24.27	86	1.18
11	1907090177	1	75	女	30.17	120	1.51
12	1907010350	1	67	女	26.85	101	1.32
13	1906120111	1	57	女	22.42	75	1.08
14	1906100298	1	72	女	29.08	114	1.45
15	1908200263	1	54	女	20.94	67	1.00
16	2002280025	1	71	女	28.35	109	1.41
17	2003260122	1	62	女	24.61	88	1.20
18	2003260240	1	68	女	27.33	104	1.35
19	2003280044	1	74	女	29.80	118	1.49
20	1905290283	1	63	女	25.14	91	1.23
21	1912180133	1	57	女	22.51	76	1.08
22	1912090127	1	76	女	30.98	125	1.55
23	1911200023	1	69	女	27.88	107	1.38
24	1911270104	1	59	女	23.26	80	1.12
25	1911130165	1	56	女	22.08	73	1.06
26	1910100304	1	59	女	23.27	80	1.12
27	1908210064	1	53	女	18.59	53	0.87
28	1903210081	1	57	男	22.25	74	1.07
29	1902210028	1	59	男	23.39	81	1.13
30	1809090083	1	70	男	28.24	109	1.40
31	1909090295	1	66	男	26.39	98	1.30
32	1909040260	1	60	男	23.70	82	1.15
33	1908010193	1	51	男	19.65	59	0.92
34	1907290336	1	55	男	17.17	45	0.79
35	1911110078	1	58	男	22.95	78	1.11
36	1907240306	1	54	男	20.83	66	0.99
37	1907240305	2	52	女	19.58	56	0.75
38	1907240304	2	62	女	25.43	88	1.20
39	1907220108	2	65	女	27.03	97	1.32
40	1909250145	2	61	女	24.38	82	1.12
41	1909170136	2	67	女	28.01	102	1.39
42	1910270029	2	60	女	24.26	82	1.11
43	1909160074	2	69	女	29.39	110	1.50
44	1910070063	2	68	女	28.56	105	1.44
45	1911250027	2	56	女	21.8	68	0.92
46	1911250094	2	50	女	18.25	49	0.65

47	1911250150	2	54	女	20.86	63	0.85
48	1911180149	2	64	女	26.15	92	1.25
49	1911110173	2	60	女	23.97	80	1.09
50	1910290031	2	54	女	20.31	60	0.81
51	1811050237	2	43	女	14.07	26	0.33
52	2003050105	2	67	女	28.1	103	1.40
53	2004090055	2	67	女	28.22	104	1.41
54	2004140259	2	56	女	21.9	69	0.93
55	2004150259	2	47	女	16.77	41	0.54
56	2005050126	2	62	女	25.48	89	1.20
57	2005120287	2	72	女	30.98	119	1.62
58	1912020055	2	75	女	32.57	127	1.74
59	1809170312	2	54	女	20.76	63	0.84
60	1904160241	2	76	女	33.45	132	1.81
61	1911060103	2	61	女	24.81	85	1.15
62	1911090170	2	60	女	23.86	80	1.08
63	1907220378	2	68	女	28.89	107	1.46
64	1911130082	2	60	女	23.97	80	1.09
65	1911230051	2	67	男	28	102	1.39
66	2005070278	2	68	男	28.42	105	1.43
67	2004250127	2	62	男	25.45	88	1.20
68	1811140195	2	61	男	24.38	82	1.12
69	2006010277	2	61	男	24.39	83	1.12
70	2005270170	2	66	男	27.32	99	1.34
71	2005170132	2	64	男	26.2	92	1.26
72	1912170245	2	58	男	23.14	76	1.02
73	1912200149	2	65	男	27.1	97	1.32
74	1912230365	2	58	男	22.96	75	1.01
75	1910060048	3	71	女	28.86	106	1.45
76	1902160143	3	50	女	17.09	34	0.46
77	1809190112	3	69	女	27.45	98	1.33
78	1907070061	3	73	女	29.74	112	1.53
79	1901090115	3	66	女	26.02	89	1.21
80	1912300164	3	61	女	23	70	0.96
81	1811120144	3	60	女	22.77	69	0.94
82	2005310122	3	77	女	32.1	126	1.73
83	1912240043	3	57	女	20.83	57	0.77
84	2001130419	3	69	女	27.81	100	1.36
85	2003050100	3	68	女	27	95	1.30
86	2003190158	3	64	女	24.95	82	1.12
87	2003190211	3	51	女	17.84	38	0.52
88	2003240209	3	74	女	30.41	116	1.58
89	2003270052	3	58	女	21.8	63	0.86
90	2003310219	3	66	女	26.08	89	1.22
91	2001060084	3	47	女	15.43	24	0.32
92	2003050030	3	69	女	27.52	98	1.34
93	2003270015	3	79	女	33.13	133	1.81

94	2004100114	3	65	女	25.15	83	1.14
95	1902270237	3	62	女	23.74	75	1.02
96	1810120189	3	76	女	31.36	122	1.66
97	1904010326	3	71	女	28.78	106	1.45
98	1807148432	3	70	女	28.11	102	1.39
99	1905090264	3	64	女	25.08	83	1.13
100	1905130058	3	66	女	25.86	88	1.20
101	1905140039	3	72	女	29.07	108	1.47
102	1905140229	3	60	女	22.77	69	0.94
103	1905150031	3	72	女	29.09	108	1.47
104	1905220183	3	66	女	25.7	87	1.19
105	1901020150	3	66	男	25.81	88	1.19
106	1810110142	3	60	男	22.91	70	0.95
107	2005260046	3	63	男	24.06	77	1.05
108	2006070052	3	73	男	29.89	113	1.54
109	1904160228	3	47	男	15.32	23	0.31
110	1902140289	3	61	男	23.15	71	0.97
111	2004130034	3	64	男	25.01	83	1.13
112	2004200172	3	61	男	22.98	70	0.96
113	1911280059	4	69	女	28.86	109	1.46
114	1911110409	4	54	女	19.56	58	0.93
115	1911110202	4	58	女	22.09	72	1.07
116	1809090062	4	76	女	33.63	135	1.73
117	1903200088	4	61	女	24.09	83	1.19
118	1902130161	4	61	女	24.11	83	1.19
119	1812120031	4	65	女	26.85	98	1.34
120	1810160073	4	66	女	27.2	100	1.36
121	1905220173	4	69	女	28.87	109	1.46
122	1901020149	4	62	女	24.8	87	1.23
123	1810110121	4	72	女	30.94	120	1.58
124	2005260044	4	73	女	31.83	125	1.63
125	2006070059	4	50	女	17.46	46	0.81
126	1904160225	4	72	女	31.09	121	1.59
127	1902140277	4	73	女	31.81	125	1.63
128	2004130024	4	64	女	25.71	92	1.28
129	2004200151	4	66	女	26.96	98	1.35
130	1911280034	4	66	女	27.31	100	1.37
131	1911110123	4	63	女	25.4	90	1.26
132	1911110122	4	56	女	21.16	67	1.02
133	1809090452	4	55	女	20.02	60	0.95
134	1903200076	4	55	女	20.35	62	0.97
135	1902130153	4	57	女	21.7	70	1.05
136	1811120134	4	65	女	26.34	95	1.31
137	2005310154	4	59	女	23.02	77	1.12
138	1912240031	4	65	女	26.5	96	1.32
139	2001130421	4	60	女	23.42	79	1.15
140	2003050112	4	57	女	21.39	68	1.03

141	2003190152	4	59	女	22.96	76	1.12
142	2003190121	4	57	男	21.62	69	1.04
143	1905290223	4	61	男	24.26	84	1.20
144	1912180113	4	66	男	27.05	99	1.35
145	1912090221	4	58	男	22.33	73	1.08
146	19112000223	4	55	男	20.1	61	0.96
147	1911270123	4	75	男	32.67	130	1.68
148	1911130264	4	64	男	25.83	92	1.28
149	1910100204	4	69	男	28.83	109	1.46

热痛阈值差值24h	热痛阈值差值48h	超C术前	超C3	超C24	超C48	ASA分级
4.39	4.12	2.65	27.24	57.43	151.08	III
2.83	2.75	1.78	21.91	49.05	134.97	II
3.45	3.30	2.13	24.04	52.40	141.41	II
2.21	2.20	1.43	19.78	45.70	128.53	II
4.11	3.87	2.49	26.27	55.90	148.14	III
3.44	3.29	2.12	23.99	52.32	141.26	II
2.34	2.32	1.51	20.23	46.41	129.90	II
1.79	1.83	1.20	18.36	43.46	124.23	II
4.77	4.46	2.87	28.55	59.48	155.03	III
3.08	2.97	1.92	22.76	50.39	137.54	II
4.38	4.12	2.65	27.21	57.38	150.99	III
3.65	3.47	2.24	24.71	53.45	143.42	III
2.67	2.61	1.69	21.36	48.19	133.31	II
4.14	3.90	2.51	26.39	56.09	148.50	III
2.34	2.32	1.51	20.24	46.43	129.94	II
3.98	3.76	2.42	25.84	55.23	146.85	II
3.15	3.03	1.96	23.02	50.79	138.31	II
3.76	3.56	2.30	25.07	54.02	144.52	III
4.30	4.04	2.60	26.94	56.95	150.15	III
3.27	3.14	2.03	23.41	51.41	139.52	II
2.69	2.62	1.70	21.43	48.29	133.52	II
4.56	4.27	2.75	27.82	58.34	152.84	III
3.88	3.67	2.37	25.48	54.67	145.77	II
2.85	2.77	1.79	21.99	49.18	135.22	II
2.59	2.54	1.65	21.10	47.78	132.53	II
2.86	2.77	1.80	22.00	49.20	135.25	II
1.82	1.86	1.22	18.47	43.64	124.57	II
2.63	2.58	1.67	21.24	47.99	132.94	II
2.88	2.80	1.81	22.09	49.34	135.53	II
3.96	3.74	2.41	25.76	55.10	146.60	III
3.55	3.38	2.18	24.36	52.89	142.36	II
2.95	2.86	1.85	22.33	49.70	136.23	II
2.06	2.07	1.35	19.27	44.90	126.99	I
1.51	1.58	1.04	17.39	41.95	121.33	I
2.79	2.71	1.76	21.76	48.82	134.52	II
2.32	2.30	1.49	20.16	46.30	129.68	II
2.12	1.98	1.36	7.70	26.53	87.27	I
2.11	3.12	2.14	12.17	32.95	97.92	II
3.21	3.44	2.35	13.40	34.71	100.84	II
3.51	2.92	2.00	11.37	31.80	96.01	II
3.02	3.63	2.49	14.15	35.79	102.64	II
3.70	2.90	1.98	11.28	31.67	95.80	II
2.99	3.90	2.67	15.20	37.30	105.14	II
3.96	3.74	2.56	14.57	36.39	103.63	III
3.80	2.42	1.66	9.40	28.96	91.31	II
2.53	1.72	1.18	6.69	25.07	84.85	II

1.86	2.23	1.53	8.68	27.93	89.60	II
2.35	3.27	2.24	12.72	33.74	99.24	III
3.35	2.84	1.95	11.06	31.35	95.26	II
2.94	2.13	1.46	8.26	27.34	88.61	III
2.25	0.91	0.63	3.50	20.49	77.24	II
1.08	3.65	2.50	14.22	35.89	102.80	III
3.72	3.67	2.51	14.31	36.02	103.02	II
3.74	2.43	1.67	9.47	29.07	91.49	II
2.55	1.43	0.99	5.55	23.44	82.15	III
1.58	3.13	2.15	12.21	33.00	98.01	II
3.22	4.21	2.88	16.42	39.05	108.04	III
4.26	4.52	3.09	17.64	40.79	110.94	II
4.56	2.21	1.52	8.61	27.83	89.43	II
2.34	4.69	3.21	18.30	41.75	112.53	III
4.72	3.00	2.06	11.70	32.27	96.80	II
3.10	2.82	1.93	10.97	31.23	95.07	II
2.92	3.80	2.60	14.82	36.75	104.23	II
3.86	2.84	1.95	11.06	31.34	95.26	III
2.94	3.63	2.48	14.14	35.78	102.61	II
3.70	3.71	2.54	14.46	36.23	103.37	III
3.78	3.13	2.14	12.19	32.97	97.96	II
3.22	2.92	2.00	11.37	31.80	96.01	II
3.02	2.92	2.00	11.38	31.81	96.04	III
3.02	3.49	2.39	13.62	35.03	101.37	II
3.57	3.27	2.24	12.76	33.79	99.33	III
3.36	2.68	1.84	10.43	30.44	93.76	II
2.78	3.45	2.36	13.45	34.78	100.96	II
3.53	2.64	1.81	10.29	30.24	93.43	III
2.64	2.40	2.43	14.85	53.57	104.30	I
0.62	0.77	1.20	5.16	38.87	81.21	I
2.40	2.20	2.29	25.86	51.81	145.88	II
2.80	2.52	2.53	27.59	54.67	150.54	II
2.16	2.01	2.14	24.78	50.03	142.97	II
1.64	1.59	1.82	22.50	46.26	136.81	II
1.60	1.56	1.79	22.32	45.97	136.34	II
3.20	2.85	2.77	29.38	57.62	155.36	II
1.27	1.29	1.59	20.86	43.55	132.39	II
2.46	2.25	2.32	26.13	52.26	146.61	II
2.32	2.14	2.24	25.52	51.24	144.96	I
1.97	1.86	2.02	23.97	48.69	140.78	II
0.75	0.87	1.27	18.59	39.80	126.28	II
2.91	2.61	2.60	28.10	55.51	151.91	II
1.43	1.42	1.69	21.59	44.76	134.36	II
2.17	2.01	2.14	24.83	50.10	143.09	III
0.34	0.54	1.02	16.76	36.79	121.37	II
2.41	2.21	2.29	25.91	51.89	146.02	II
3.38	2.99	2.88	30.16	58.91	157.47	II

2.01	1.88	2.04	24.12	48.93	141.18	III
1.76	1.69	1.89	23.05	47.17	138.31	II
3.07	2.75	2.70	28.82	56.69	153.85	II
2.63	2.39	2.42	26.86	53.46	148.58	III
2.52	2.30	2.35	26.36	52.63	147.22	II
1.99	1.88	2.04	24.07	48.85	141.05	II
2.13	1.98	2.12	24.66	49.82	142.64	II
2.68	2.43	2.46	27.08	53.83	149.18	III
1.60	1.56	1.79	22.32	45.96	136.33	II
2.68	2.43	2.46	27.10	53.86	149.22	II
2.10	1.96	2.10	24.54	49.63	142.32	II
2.12	1.98	2.11	24.62	49.76	142.53	III
1.62	1.58	1.81	22.43	46.14	136.62	II
1.82	1.73	1.93	23.29	47.57	138.96	III
2.82	2.54	2.54	27.71	54.85	150.85	II
0.32	0.52	1.01	16.68	36.66	121.15	III
1.66	1.61	1.83	22.61	46.44	137.12	II
1.98	1.87	2.03	24.02	48.77	140.91	II
1.63	1.59	1.82	22.48	46.23	136.77	III
2.71	2.47	2.59	15.28	38.38	102.13	II
1.32	1.17	1.47	9.06	26.57	87.32	II
1.70	1.52	1.77	10.76	29.79	91.36	II
3.43	3.15	3.17	18.48	44.44	109.74	II
2.00	1.80	2.02	12.09	32.33	94.54	II
2.00	1.81	2.02	12.10	32.35	94.57	I
2.41	2.19	2.35	13.94	35.83	98.93	II
2.46	2.24	2.39	14.17	36.27	99.49	II
2.72	2.48	2.60	15.29	38.40	102.16	II
2.10	1.90	2.10	12.56	33.22	95.66	III
3.03	2.77	2.85	16.67	41.02	105.45	II
3.16	2.89	2.96	17.27	42.15	106.87	III
1.00	0.87	1.21	7.66	23.92	83.98	II
3.05	2.79	2.87	16.77	41.21	105.69	III
3.16	2.89	2.95	17.26	42.12	106.83	II
2.24	2.03	2.21	13.18	34.39	97.13	II
2.43	2.21	2.36	14.01	35.96	99.10	III
2.48	2.26	2.41	14.25	36.41	99.67	II
2.20	1.99	2.18	12.97	33.99	96.63	II
1.56	1.39	1.66	10.13	28.60	89.86	III
1.39	1.23	1.52	9.37	27.16	88.05	III
1.44	1.28	1.56	9.59	27.58	88.58	II
1.64	1.47	1.73	10.49	29.29	90.72	III
2.34	2.12	2.29	13.60	35.19	98.13	II
1.84	1.65	1.89	11.37	30.96	92.83	III
2.36	2.14	2.31	13.70	35.39	98.38	II
1.90	1.71	1.94	11.65	31.48	93.48	II
1.59	1.42	1.69	10.28	28.90	90.24	III

1.83	1.65	1.88	11.34	30.89	92.74	II
1.63	1.46	1.72	10.44	29.19	90.60	III
2.02	1.83	2.04	12.21	32.55	94.82	II
2.44	2.22	2.38	14.07	36.08	99.26	III
1.73	1.56	1.80	10.92	30.09	91.74	II
1.40	1.24	1.53	9.42	27.26	88.18	III
3.29	3.01	3.06	17.83	43.22	108.21	II
2.26	2.05	2.23	13.25	34.53	97.30	III
2.71	2.47	2.59	15.27	38.35	102.09	II

术前动态	术前静态	HSS	术后6小时静	术后12小时静	术后24h静态	术后48h	术后6h动态
8	8	48	5	5	5	3	8
6	6	48	3	3	3	2	6
7	7	39	4	4	4	2	7
5	5	43	3	3	3	1	6
7	7	36	5	5	4	3	8
7	7	46	4	4	4	2	7
5	5	43	3	3	3	1	6
4	4	37	2	3	2	1	5
8	8	34	5	5	5	4	9
6	6	50	4	4	4	2	7
8	8	41	5	5	5	3	8
7	7	48	4	4	4	3	7
5	5	44	3	3	3	2	6
7	7	39	5	5	4	3	8
5	5	46	3	3	3	1	6
7	7	37	5	4	4	3	8
6	6	45	4	4	4	2	7
7	7	41	4	4	4	3	8
8	8	44	5	5	5	3	8
6	6	47	4	4	4	2	7
6	6	42	3	3	3	2	6
8	8	39	5	5	5	4	9
7	7	49	5	4	4	3	8
6	6	45	3	3	3	2	6
5	5	40	3	3	3	2	6
6	6	38	3	3	3	2	6
4	4	40	2	3	2	1	5
5	5	34	3	3	3	2	6
6	6	38	4	3	3	2	7
7	7	40	5	4	4	3	8
7	7	45	4	4	4	3	7
6	6	43	4	4	3	2	7
5	5	40	3	3	3	1	5
4	4	35	2	2	2	0	5
6	6	33	3	3	3	2	6
5	5	39	3	3	3	1	6
5	5	37	2	1	1	1	5
7	7	36	3	2	2	2	6
7	7	41	3	3	2	2	7
6	6	42	3	2	2	2	6
7	7	40	3	3	3	3	7
6	6	43	2	2	2	2	6
8	8	40	3	3	3	3	7
7	7	44	3	3	3	3	7
6	6	44	2	2	1	1	5
5	5	38	1	1	1	0	4

5	5	34	2	2	1	1	5
7	7	37	3	2	2	2	6
6	6	41	2	2	2	2	6
5	5	39	2	2	1	1	5
4	4	36	1	1	0	1	3
7	7	31	3	3	3	3	7
7	7	43	3	3	3	3	7
6	6	43	2	2	1	1	5
4	4	38	1	1	0	0	4
7	7	33	3	2	2	2	6
8	8	41	4	3	3	3	8
8	8	46	4	4	4	4	8
5	5	47	2	2	1	1	5
9	9	37	4	4	4	4	8
6	6	48	3	2	2	2	6
6	6	40	2	2	2	2	6
7	7	39	3	3	3	3	7
6	6	44	2	2	2	2	6
7	7	39	3	3	3	3	7
7	7	43	3	3	3	3	7
7	7	44	3	2	2	2	6
6	6	41	3	2	2	2	6
6	6	40	3	2	2	2	6
7	7	40	3	3	3	2	7
7	7	43	3	2	2	2	6
6	6	42	2	2	2	1	6
7	7	39	3	3	2	2	7
6	6	42	2	2	2	1	5
8	8	39	4	4	4	3	7
4	4	44	1	1	1	0	4
7	7	31	4	3	3	2	7
8	8	42	4	4	4	3	7
7	7	44	3	3	3	2	6
6	6	40	3	2	2	2	6
6	6	37	3	2	2	1	5
9	9	37	5	4	4	3	8
5	5	47	2	2	2	1	5
7	7	35	4	3	3	2	7
7	7	42	3	3	3	2	7
7	7	41	3	3	3	2	6
5	5	39	1	2	1	0	4
8	8	32	4	4	4	3	8
6	6	45	2	2	2	1	5
7	7	36	3	3	3	2	6
4	4	40	1	1	1	0	3
7	7	29	4	3	3	2	7
9	9	42	5	4	5	4	8

7	7	48	3	3	3	2	6
6	6	39	3	3	3	2	6
8	8	38	4	4	4	3	8
8	8	46	4	4	4	3	7
7	7	43	4	3	4	3	7
7	7	42	3	3	3	2	6
7	7	39	3	3	3	2	6
8	8	40	4	4	4	3	7
6	6	43	3	2	2	1	5
8	8	37	4	4	4	3	7
7	7	43	3	3	3	2	6
7	7	40	3	3	3	2	6
6	6	40	3	2	2	2	6
6	6	37	3	3	3	2	6
8	8	38	4	4	4	3	7
4	4	44	1	1	1	0	3
6	6	29	3	3	2	2	6
7	7	37	3	3	3	2	6
6	6	39	3	2	2	2	6
7	7	37	3	2	2	2	6
5	5	43	1	1	1	1	4
5	5	33	1	1	1	1	5
9	9	36	4	3	3	3	7
6	6	48	2	2	1	1	5
6	6	38	2	2	1	1	5
7	7	38	2	2	2	2	6
7	7	41	2	2	2	2	6
7	7	41	3	2	2	2	6
6	6	43	2	2	2	2	5
8	8	39	3	3	3	3	7
8	8	45	3	3	3	3	7
4	4	46	0	1	0	0	3
8	8	31	3	3	3	3	7
8	8	45	3	3	3	3	7
6	6	46	2	2	2	2	5
7	7	40	2	2	2	2	6
7	7	41	2	2	2	2	6
6	6	41	2	2	2	2	5
5	5	39	1	1	1	1	4
5	5	35	1	1	1	1	4
5	5	34	1	1	1	1	4
5	5	34	1	1	1	1	4
7	7	36	2	2	2	2	6
6	6	40	2	2	1	1	5
7	7	37	2	2	2	2	6
6	6	41	2	2	1	1	5
5	5	37	1	1	1	1	4

6	6	35	2	2	1	1	5
5	5	37	1	1	1	1	4
6	6	35	2	2	1	2	5
7	7	38	2	2	2	2	6
6	6	41	1	1	1	1	5
5	5	36	1	1	1	1	4
8	8	34	4	3	3	3	7
7	7	47	2	2	2	2	5
7	7	40	3	2	2	2	6

术后12h动?	术后24h动?	术后48h动	6h芬太尼	12h芬太尼	24h芬太尼	48h芬太尼	第一次直腿抬
8	8	4	278.94	426.50	610.22	960.68	26.79
6	5	3	183.32	264.53	394.50	681.88	20.47
7	6	3	221.55	329.29	480.74	793.35	23.00
6	4	2	145.09	199.77	308.25	570.41	17.94
8	7	4	261.47	396.91	570.80	909.73	25.63
7	6	3	220.68	327.83	478.79	790.83	22.94
6	4	2	153.23	213.57	326.62	594.16	18.48
5	3	2	119.61	156.63	250.78	496.14	16.26
9	8	5	302.37	466.19	663.07	1028.99	28.34
7	6	3	198.61	290.43	428.99	726.46	21.48
8	8	4	278.39	425.57	608.98	959.08	26.75
7	6	3	233.47	349.49	507.65	828.12	23.79
6	5	2	173.48	247.87	372.30	653.19	19.82
8	7	4	263.61	400.55	575.64	916.00	25.78
6	4	2	153.48	213.99	327.18	594.88	18.50
8	7	4	253.83	383.97	553.57	887.46	25.13
7	6	3	203.16	298.15	439.27	739.74	21.78
8	7	4	240.03	360.59	522.44	847.23	24.22
8	7	4	273.42	417.15	597.75	944.57	26.42
7	6	3	210.31	310.25	455.39	760.57	22.25
6	5	2	174.70	249.93	375.05	656.74	19.90
9	8	4	289.36	444.16	633.73	991.07	27.48
8	7	4	247.41	373.10	539.09	868.75	24.71
6	5	3	184.84	267.11	397.93	686.32	20.57
6	5	2	168.85	240.02	361.85	639.69	19.51
6	5	3	185.02	267.41	398.33	686.84	20.58
5	4	2	121.63	160.04	255.32	502.01	16.39
6	5	2	171.26	244.11	367.30	646.73	19.67
6	5	3	186.64	270.16	401.99	691.56	20.69
8	7	4	252.33	381.43	550.18	883.09	25.03
7	6	3	227.19	338.85	493.48	809.80	23.37
7	5	3	190.80	277.21	411.38	703.70	20.97
5	4	2	135.98	184.35	287.71	543.87	17.34
5	3	1	102.38	127.43	211.89	445.88	15.12
6	5	3	180.68	260.07	388.55	674.20	20.30
5	4	2	151.96	211.42	323.75	590.45	18.40
4	3	2	92.31	126.52	306.83	454.17	17.73
6	5	3	157.98	224.36	283.75	637.95	20.62
6	5	3	175.98	251.17	416.99	688.32	21.42
6	4	3	146.20	206.81	453.51	604.99	20.10
6	5	3	187.03	267.64	393.09	719.26	21.90
6	4	3	144.89	204.86	475.93	601.32	20.04
7	6	4	202.44	290.60	390.43	762.39	22.58
7	6	3	193.14	276.74	507.21	736.36	22.17
5	4	2	117.22	163.63	488.34	523.88	18.82
4	3	1	77.43	104.36	334.29	412.53	17.07

5	4	2	106.68	147.94	253.56	494.40	18.36
6	5	3	166.08	236.42	312.92	660.62	20.98
5	4	3	141.58	199.93	433.43	592.06	19.90
5	3	2	100.57	138.83	383.72	477.28	18.09
3	2	1	30.52	34.49	300.51	281.27	15.00
6	5	3	188.03	269.13	158.40	722.06	21.95
7	6	3	189.38	271.14	477.97	725.83	22.01
5	4	2	118.33	165.30	480.70	527.01	18.87
4	2	1	60.76	79.52	336.56	365.88	16.33
6	5	3	158.53	225.18	219.74	639.49	20.65
7	6	4	220.36	317.28	418.11	812.52	23.37
8	7	4	238.22	343.89	543.55	862.51	24.16
5	4	2	105.63	146.36	579.79	491.44	18.31
8	7	4	248.03	358.50	310.77	889.96	24.59
6	5	3	151.05	214.04	599.69	618.57	20.32
5	4	3	140.37	198.13	402.94	588.67	19.85
7	6	3	196.86	282.28	381.27	746.77	22.34
5	4	3	141.57	199.91	495.88	592.03	19.90
6	5	3	186.89	267.42	383.70	718.86	21.90
7	6	3	191.56	274.39	475.64	731.94	22.10
6	5	3	158.22	224.72	485.13	638.63	20.63
6	4	3	146.18	206.78	417.48	604.94	20.10
6	4	3	146.36	207.05	393.06	605.43	20.11
6	5	3	179.23	256.02	393.41	697.43	21.56
6	5	3	166.62	237.23	460.11	662.13	21.00
5	4	2	132.34	186.16	434.52	566.20	19.49
6	5	3	176.71	252.26	364.97	690.38	21.45
5	4	2	130.29	183.11	455.00	560.46	19.40
7	6	4	198.07	304.76	495.30	731.85	22.73
4	2	1	66.69	97.30	189.44	361.56	14.03
6	5	3	182.32	279.89	458.63	687.46	21.68
7	6	4	207.85	320.21	518.07	759.42	23.37
6	5	3	166.36	254.69	421.48	642.48	20.63
5	4	2	132.68	201.49	343.05	547.53	18.40
5	4	2	130.09	197.42	337.05	540.26	18.23
8	6	4	234.20	361.82	579.42	833.69	25.12
5	3	2	108.48	163.28	286.71	479.32	16.80
7	5	3	186.32	286.20	467.94	698.72	21.95
7	5	3	177.26	271.89	446.84	673.19	21.35
6	5	3	154.39	235.79	393.61	608.74	19.84
4	3	1	75.00	110.42	208.79	384.98	14.58
8	6	4	215.36	332.06	535.55	780.58	23.87
5	4	2	119.25	180.29	311.81	509.70	17.51
6	5	3	167.04	255.75	423.05	644.38	20.67
3	2	1	48.09	67.92	146.14	309.14	12.80
7	5	3	183.06	281.06	460.36	689.56	21.73
8	7	4	245.76	380.07	606.33	866.27	25.88

6	5	3	156.58	239.25	398.72	614.92	19.98
6	4	2	140.85	214.40	362.08	570.57	18.94
8	6	4	225.94	348.76	560.17	810.39	24.57
7	6	4	197.10	303.23	493.05	729.12	22.66
7	5	3	189.67	291.49	475.73	708.16	22.17
6	5	3	155.85	238.09	397.02	612.86	19.93
6	5	3	164.56	251.84	417.27	637.39	20.51
7	6	4	200.37	308.39	500.65	738.32	22.88
5	4	2	130.05	197.35	336.95	540.14	18.23
7	6	4	200.60	308.75	501.18	738.97	22.89
6	5	3	162.82	249.09	413.23	632.50	20.39
6	5	3	163.99	250.95	415.96	635.80	20.47
5	4	2	131.64	199.85	340.64	544.61	18.33
6	4	3	144.43	220.06	370.43	580.67	19.18
7	6	4	209.53	322.85	521.97	764.14	23.48
3	2	1	46.90	66.04	143.36	305.77	12.72
5	4	2	134.35	204.13	346.95	552.25	18.51
6	5	3	155.12	236.94	395.31	610.80	19.89
5	4	2	132.43	201.10	342.48	546.84	18.38
5	4	3	159.62	190.76	358.60	586.63	19.78
3	2	1	49.91	80.02	198.75	338.13	15.02
4	3	2	79.85	110.24	242.38	405.95	16.32
7	5	4	215.96	247.64	440.69	714.24	22.22
4	3	2	103.39	134.00	276.68	459.27	17.34
4	3	2	103.60	134.21	276.97	459.73	17.35
5	4	3	135.92	166.84	324.06	532.93	18.75
5	4	3	140.05	171.01	330.08	542.29	18.93
5	4	3	159.82	190.97	358.89	587.08	19.79
4	3	2	111.71	142.40	288.80	478.12	17.70
6	5	4	184.16	215.54	394.36	642.22	20.84
6	5	4	194.67	226.14	409.67	666.01	21.30
2	2	1	25.23	55.10	162.79	282.23	13.95
6	5	4	185.95	217.34	396.96	646.25	20.92
6	5	4	194.42	225.90	409.31	665.46	21.29
5	4	2	122.55	153.34	304.59	502.66	18.17
5	4	3	137.18	168.11	325.90	535.80	18.80
5	4	3	141.37	172.34	332.01	545.30	18.99
5	3	2	118.89	149.65	299.26	494.38	18.01
3	2	1	68.77	99.05	226.23	380.84	15.84
3	2	1	55.36	85.52	206.70	350.48	15.25
3	2	1	59.29	89.48	212.42	359.37	15.42
4	3	1	75.14	105.48	235.51	395.28	16.11
5	4	2	129.96	160.82	315.39	519.45	18.49
4	3	2	90.71	121.20	258.20	430.55	16.79
5	4	3	131.82	162.70	318.10	523.66	18.57
4	3	2	95.54	126.07	265.23	441.48	17.00
3	3	1	71.53	101.84	230.25	387.10	15.96

4	3	2	90.08	120.56	257.28	429.11	16.76
4	3	1	74.24	104.57	234.20	393.23	16.07
4	3	2	105.44	136.07	279.67	463.92	17.43
5	4	3	138.32	169.27	327.57	538.39	18.85
4	3	2	82.64	113.05	246.44	412.26	16.44
3	2	1	56.30	86.47	208.07	352.61	15.29
7	5	4	204.63	236.21	424.19	688.59	21.73
5	4	2	123.85	154.66	306.49	505.62	18.23
5	4	3	159.34	190.48	358.19	585.99	19.77

第一次抢救	术后24hROM	术后48hROM	术后72hROM	PONV	过度镇静	晕眩	尿潴留
102.56	56.54	70.00	83.25	0	0	0	0
85.81	43.94	54.63	67.99	0	0	0	0
92.51	48.98	60.78	74.09	0	0	0	1
79.12	38.90	48.49	61.89	0	0	0	0
99.50	54.24	67.19	80.46	0	0	1	0
92.36	48.86	60.64	73.96	0	0	0	0
80.54	39.98	49.80	63.19	1	0	0	0
74.66	35.55	44.39	57.83	0	0	0	0
106.66	59.63	73.77	86.99	1	0	0	0
88.49	45.96	57.09	70.43	0	0	0	0
102.46	56.47	69.92	83.16	1	0	0	0
94.60	50.55	62.70	76.00	0	0	0	0
84.09	42.64	53.05	66.42	0	0	0	0
99.87	54.52	67.54	80.81	1	0	0	0
80.59	40.01	49.84	63.23	0	0	0	0
98.16	53.23	65.97	79.24	1	0	0	0
89.29	46.56	57.82	71.16	0	0	0	0
95.74	51.41	63.75	77.04	1	0	0	0
101.59	55.81	69.12	82.37	0	0	0	0
90.54	47.50	58.97	72.30	0	0	0	0
84.30	42.80	53.25	66.62	0	0	0	0
104.38	57.92	71.68	84.91	0	0	0	0
97.04	52.39	64.94	78.22	1	0	0	0
86.08	44.14	54.88	68.24	0	0	0	0
83.28	42.03	52.31	65.68	0	0	0	0
86.11	44.16	54.91	68.26	0	0	0	0
75.01	35.81	44.72	58.15	0	0	0	0
83.70	42.35	52.70	66.07	0	0	0	0
86.39	44.38	55.17	68.52	0	0	0	0
97.90	53.03	65.73	79.00	0	0	0	0
93.50	49.72	61.69	74.99	1	0	0	0
87.12	44.93	55.84	69.19	0	0	0	0
77.52	37.70	47.02	60.44	0	0	0	0
71.64	33.27	41.62	55.08	0	0	0	0
85.35	43.59	54.21	67.57	0	0	0	0
80.32	39.81	49.59	62.99	0	0	0	0
94.00	34.73	48.40	65.41	0	0	0	0
110.86	45.54	59.43	76.43	0	0	0	0
115.49	48.50	62.45	79.45	0	0	0	0
107.84	43.60	57.45	74.45	0	0	0	0
118.33	50.32	64.31	81.30	0	0	0	0
107.50	43.39	57.23	74.23	0	0	0	0
122.29	52.86	66.90	83.89	0	0	0	1
119.90	51.33	65.34	82.33	0	0	0	0
100.40	38.83	52.58	69.59	0	0	0	0
90.18	32.28	45.90	62.91	0	0	0	0

97.69	37.10	50.81	67.82	0	0	0	0
112.95	46.87	60.79	77.79	0	0	0	0
106.65	42.84	56.67	73.68	0	0	0	0
96.12	36.09	49.78	66.80	1	0	0	0
78.13	24.56	38.02	55.05	0	0	0	0
118.58	50.49	64.48	81.47	0	0	0	0
118.93	50.71	64.70	81.70	1	0	0	0
100.68	39.01	52.77	69.78	0	0	0	0
85.89	29.54	43.10	60.12	1	0	0	0
111.01	45.63	59.52	76.52	0	0	0	0
126.89	55.81	69.91	86.89	0	0	0	0
131.47	58.75	72.91	89.89	1	0	0	0
97.42	36.92	50.63	67.64	0	0	0	0
133.99	60.36	74.55	91.53	0	0	0	0
109.09	44.40	58.26	75.27	1	0	0	0
106.34	42.64	56.47	73.47	0	0	0	0
120.85	51.94	65.96	82.95	0	0	0	0
106.65	42.84	56.67	73.67	1	0	0	0
118.29	50.30	64.28	81.28	0	0	0	0
119.49	51.07	65.07	82.06	0	0	0	0
110.93	45.58	59.47	76.47	0	0	0	0
107.83	43.60	57.45	74.45	0	0	0	0
107.88	43.63	57.48	74.48	0	0	1	0
116.32	49.04	63.00	79.99	0	0	0	0
113.08	46.96	60.88	77.88	0	0	0	0
104.28	41.32	55.12	72.13	0	0	0	0
115.68	48.62	62.57	79.57	0	0	0	0
103.75	40.98	54.78	71.78	0	0	0	0
116.98	51.16	62.28	81.70	0	0	0	0
83.75	25.39	37.93	55.53	0	0	0	1
113.00	48.07	59.36	78.56	0	0	0	0
119.46	53.08	64.10	83.64	0	0	0	0
108.96	44.94	56.41	75.38	0	0	0	1
100.44	38.33	50.16	68.67	0	0	0	0
99.79	37.82	49.68	68.16	0	0	0	0
126.12	58.25	68.98	88.89	1	0	0	0
94.32	33.58	45.67	63.85	0	0	0	0
114.01	48.85	60.10	79.36	0	0	0	0
111.72	47.08	58.43	77.55	1	0	0	0
105.94	42.59	54.19	73.00	0	0	1	0
85.86	27.02	39.47	57.18	0	0	0	0
121.36	54.55	65.49	85.14	0	0	0	0
97.05	35.70	47.67	66.00	1	0	0	0
109.13	45.07	56.53	75.52	0	0	0	1
79.05	21.74	34.48	51.82	0	0	0	0
113.19	48.22	59.50	78.71	0	0	0	0
129.05	60.52	71.13	91.20	1	1	0	0

106.49	43.02	54.59	73.43	0	0	0	1
102.51	39.93	51.68	70.30	0	0	0	0
124.03	56.63	67.45	87.25	1	0	0	0
116.74	50.97	62.10	81.50	0	0	0	0
114.86	49.51	60.73	80.02	0	0	0	0
106.31	42.88	54.46	73.29	0	0	0	0
108.51	44.59	56.07	75.02	0	1	0	0
117.56	51.61	62.71	82.15	0	0	0	0
99.78	37.82	49.67	68.15	0	0	0	0
117.62	51.66	62.75	82.20	0	0	0	0
108.07	44.25	55.75	74.68	0	0	0	0
108.36	44.48	55.97	74.91	0	0	0	0
100.18	38.13	49.97	68.46	0	0	0	0
103.42	40.64	52.34	71.01	0	0	0	0
119.88	53.41	64.41	83.98	0	0	0	0
78.75	21.50	34.26	51.59	0	0	0	0
100.87	38.66	50.47	69.00	0	0	0	0
106.12	42.74	54.32	73.14	0	0	0	0
100.38	38.28	50.11	68.62	0	0	0	0
213.22	52.62	65.14	82.34	0	0	0	0
188.46	35.26	49.17	64.16	0	0	0	0
195.22	40.00	53.53	69.12	0	0	0	0
225.93	61.54	73.34	91.67	0	0	0	0
200.53	43.72	56.95	73.02	0	0	0	0
200.57	43.75	56.98	73.05	0	0	0	0
207.87	48.87	61.69	78.41	0	0	0	0
208.80	49.52	62.29	79.09	0	0	0	0
213.26	52.65	65.17	82.37	0	0	0	0
202.41	45.04	58.17	74.40	0	0	0	0
218.76	56.50	68.71	86.40	0	0	0	0
221.13	58.17	70.24	88.15	0	0	0	0
182.89	31.35	45.58	60.07	0	0	0	0
219.16	56.79	68.97	86.70	0	0	0	0
221.07	58.13	70.20	88.10	0	0	0	0
204.85	46.75	59.74	76.19	0	0	0	0
208.15	49.07	61.87	78.62	0	1	0	0
209.10	49.73	62.48	79.31	0	0	1	0
204.03	46.18	59.21	75.59	0	0	0	0
192.71	38.24	51.91	67.28	0	0	0	0
189.69	36.12	49.96	65.06	0	1	0	0
190.57	36.74	50.54	65.71	0	0	0	0
194.15	39.25	52.84	68.34	0	0	0	1
206.52	47.93	60.82	77.42	0	0	0	0
197.67	41.72	55.11	70.92	0	0	1	0
206.94	48.22	61.09	77.73	0	0	0	0
198.76	42.48	55.81	71.72	0	0	0	0
193.34	38.68	52.32	67.74	0	0	0	1

197.52	41.62	55.02	70.81	0	0	0	0
193.95	39.11	52.71	68.19	0	0	0	0
200.99	44.05	57.25	73.36	0	0	0	0
208.41	49.25	62.04	78.81	0	0	0	1
195.84	40.44	53.93	69.58	0	0	0	0
189.90	36.27	50.10	65.21	0	0	0	0
223.38	59.74	71.69	89.80	0	0	0	0
205.15	46.96	59.93	76.41	0	0	0	0
213.15	52.58	65.10	82.29	0	0	0	0

深静脉血栓	消化道出血
0	0
0	0
0	0
0	0
0	0
0	0
0	0
0	0
0	0
0	0
1	0
0	0
0	0
0	0
0	0
0	0
0	0
0	0
0	0
0	0
0	0
0	0
0	0
0	0
0	0
0	0
0	0
0	0
0	0
0	0
0	0
0	0
0	0
0	0
0	0
0	0
0	0
0	0
0	0
0	0
0	0
1	0
0	0
0	0
0	0
0	0

0	0
0	0
0	0
0	0
0	0
0	0
0	0
0	0
0	0
0	0
0	0
0	0
0	0
0	0
0	0
0	0
0	0
0	0
0	0
0	0
0	0
0	0
0	0
0	0
0	0
0	0
0	0
0	0
0	0
0	0
0	0
0	0
0	0
0	0
0	0
0	0
0	0
0	0
0	0
0	0
0	0
0	0
0	0
0	0
0	0
0	0

0	0
0	0
0	0
0	0
0	0
1	0
0	0
0	0
0	0
0	0
0	0
1	0
0	0
0	0
0	0
0	0
0	0
0	0
0	0
0	0
0	0
0	0
0	0
0	0
0	0
0	0
0	0
0	0
0	0
0	0
0	0
0	0
0	0
0	0
0	0
0	0
0	0
0	0
0	0
0	0
0	0
0	0
0	0
0	0
0	0
0	0
0	0

0	0
0	0
0	0
0	0
0	0
0	0
0	0
0	0
0	0
